# Disrupted Biotensegrity in the Fiber Cellular Fascial Network and Neuroma Microenvironment: A Conceptual Framework for “Phantom Limb Pain”

**DOI:** 10.3390/ijms26178161

**Published:** 2025-08-22

**Authors:** Shiloh Plaut

**Affiliations:** Department of Basic and Clinical Sciences, University of Nicosia Medical School, Nicosia 2408, Cyprus; shilowhale@gmail.com

**Keywords:** biotensegrity, fascia, fascial armoring, fibromyalgia, medically unexplained symptoms, theoretical model, myofibroblasts, myofascial chains, phantom pain, psychosomatic medicine

## Abstract

Among the leading etiologies of limb amputations are diabetes mellitus, alongside trauma and peripheral vascular disease conditions, whose complications are major indications for surgery, which can subsequently elicit chronic refractory postamputation pain. ‘Phantom limb pain’ (PLP) denotes pain that is perceived as occurring in an absent part of the limb following amputation. Even though it is a relatively common complication among amputees—with an estimated prevalence as high as ~80 percent—the underlying mechanisms of this puzzling condition remain poorly understood. Current theories predominantly emphasize the role of the nervous system and neuropsychopathology in the development of PLP. However, these neurocentric explanations are disputed and have not yet been translated into effective treatments or a definitive cure for the condition, nor have several notable anomalies been settled, which has prompted researchers to call for the exploration of alternative theories. The aim of this paper is to offer an alternative mechanical mechanism for explaining PLP and spontaneous phantom sensations. This work introduces a theoretical model for the mechanism of PLP, drawing on a recent study that proposed this model to explain fibromyalgia-type psychosomatic syndromes as disorders driven by overactive soft tissue myofibroblasts. The manuscript proposes a shift from purely neurocentric models of PLP to a framework where the extracellular matrix and connective tissue, specifically myofascial tissue and inflammatory myofibroblasts—which are often overlooked in research—take part in its pathogenesis. In this suggested model, surgical interventions disrupt the biomechanical stability of the fascio-musculoskeletal biotensegrity-like system, thus acting as a contributing factor in the chronic pain manifestation. The term ‘biotensegrity’ refers to the dynamic biomechanical behavior of a living system that is stabilized by compressive and tensile force elements, a characteristic quality of myofascial tissue. In this framework, abnormal extracellular matrix remodeling, driven by overactive peripheral myofibroblasts, and the concomitant mechanical effects exerted on sensory nerves embedded within the fascia and reaching the neuroma microenvironment contribute to the generation and perception of spontaneous PLP and phantom sensations. The interplay between abnormal extracellular matrix, the neuroma’s intrinsic excitability, as well as peripheral and central neurophysiological mechanisms, collectively provide a biophysical neuropathophysiological basis to help explain PLP. This offers a different unexplored perspective on a condition with poorly understood mechanisms.

## 1. Introduction

Phantom limb pain (PLP) is a term that refers to pain arising after limb amputation, typically perceived as occurring in the part of the body that is no longer physically present [1]. This peculiar phenomenon is extremely common among amputees and can be seen in as much as 60–80% of patients at some time following surgical amputation [2,3]. Diabetic foot complications and peripheral vascular disease, as well as severe trauma, are the main etiologies that often culminate in the need for amputations that subsequently leave the patients with life-changing consequences such as physical disability and chronic post-surgical pain, including such perplexing phantom sensations and pain. Reports of phantom tooth pain, phantom breast pain, and other persistent post-surgical pains being a consequence of various surgical interventions are documented in the literature [4,5]. Several theories for PLP’s pathophysiology exist, as with other disorders whose pathophysiology is undeciphered. Most focus on the putative role of neuropsychopathologies and the nervous system [1,6], yet the precise mechanisms that contribute to the development and maintenance of phantom pain remain largely unknown [6].

The theoretical complexity of chronic pain extends beyond PLP, as exemplified by fibromyalgia syndrome, for which neurobiological and psychological explanations are also prominent. Nevertheless, the current conceptualization of neuroscientific theories appears to leave certain gaps and discrepancies observed in this disorder unresolved, and the treatments that are derived from current theories are remarkably unsatisfactory. A recent study offered an alternative theoretical model with a neuro-mechanobiological mechanism for explaining psychosomatic chronic widespread pain, offering a pathogenesis for fibromyalgia-type syndromes and ‘myofascial pain syndrome’ [7]. The purpose of this paper is to apply this model to PLP.

Fibromyalgia is a chronic pain disorder of unknown etiology and poorly understood pathophysiology that typically manifests with widespread musculoskeletal discomfort and pain, hypervigilance, sympathetic imbalance, gastrointestinal disturbances, cognitive symptoms, and relentless fatigue. Its prevalence is approximately ~1–6 percent of the general population and it leads to a significant burden on the healthcare system and considerably impacts patients’ quality of life and mental health. The most accepted and widely investigated theory for “fibromyalgia” currently postulates a psycho-neurobiological explanation concerning a dysfunction of ascending and descending somatosensory processing pathways in the periphery, dorsal root ganglion, spinal cord, and brain, causing structural and functional alterations in pain pathways or a “nociplastic” malfunction. These are presumed to lead to general sensory hypersensitivity, hyperalgesia, allodynia, overactivation of the stress response, and chronic pain with no real injury or seemingly justifiable cause, while being amplified and reinforced by psychological catastrophizing. Although, there are some authors who have been questioning this thesis [8,9].

The suggested model posits an alternative explanation for functional somatic syndromes as clinical variations in one medical entity linked to hyperactivated myofibroblasts and diffuse biomechanical alterations in the extracellular matrix. This process results in increased fascial tissue rigidity, mechanical compression and subsequent neurophysiological abnormalities [7]. According to the suggested theoretical model, surgical interventions may play a critical role in influencing the progression of fibromyalgia-type psychosomatic syndromes by disrupting and modulating the homeostasis of myofascial tensegrity. The term “tensegrity” refers to a pre-stressed structure (or spatial mechanical system) that maintains stability by means of a combination of compressive and tensional force elements. Empirical evidence that was reported in the scientific medical literature indicates that certain surgical interventions alleviate fibromyalgia while other types of surgery may trigger or worsen fibromyalgia [7,10,11]. A possible explanation for the common factor among surgical interventions that modulate fibromyalgia’s disease course was hypothesized to be mechanical modulation of the myofascial tensegrity system [7].

According to the suggested model, limb amputation is likewise expected to induce new onset chronic pain by disrupting the mechanical equilibrium of the tensegrity system followed by subsequent corresponding neuropathophysiological aberrations [7]. After natural processes of myofibroblasts contractility and extracellular matrix remodeling, local interstitial and fascial cells are expected to modify the biomechanical environment around a traumatic neuroma which is formed postamputation. This neuroma, in response to surroundings extracellular matrix tensegrity forces, would serve as a substrate for nociceptive signals delivered to the dorsal root ganglion and, further downstream, the central nervous system. The aim of this paper is to offer an alternative mechanical mechanism for explaining PLP and spontaneous phantom sensations as an organic disorder involving myofibroblast-mediated biotensegrity tension and accompanying neurophysiological consequences such as pain and central neurological adaptations. This mechanism, originally suggested for “primary fibromyalgia syndrome,” is presented and applied to PLP as disorders of interconnected neurobiological and biomechanical systems. Hopefully, this work will advance our understanding of phantom pain and identify potential treatments.

The structure of this manuscript is as follows: in Section 2 current theories of PLP are reviewed; afterwards Section 3 describes the methods of the work. Section 4 will present the myofascial biotensegrity-based model for explaining PLP, with empirical findings in support of the model while Section 5 discusses the relevance of the model to PLP. Section 6 is a discussion, followed by Section 7 which concludes the manuscript.

## 2. Current Theories of Phantom Limb Pain

In this section a brief outline of current theories of PLP is given aimed not to be exhaustive but focused. Collins et al. (2018) previously reviewed the current theories of PLP in depth [6]. PLP is now believed to be a neuropathic pain or complex pain state [6] that is driven by central nervous system (CNS) abnormalities [12], and this perspective usually guides the approach to its treatment. The most posited theories for the mechanism of PLP rely on neuronal network reorganization [6]. Although PLP’s pathogenesis remains unclear, it is possible that sensitized and reorganized peripheral nerves in the limb affect input into the CNS, leading to abnormalities in central somatosensory processing systems [6,13].

Some of the theories that try to explain PLP rely on the idea of having a representation of the self within the brain (i.e., the neuromatrix) whereby life experiences continuously modify our “self” [6]. According to this notion, limb amputation causes a mismatch between cortical and peripheral body representations such that a lack of visual or sensory feedback from the missing limb exacerbates the mismatch, leading to pain with no apparent stimulus [1,6].

Central sensitization and abnormal nerve sprouting may explain PLP [1]: it is shown that overactivity of peripheral nociceptors causes permanent changes in the synaptic molecular composition and structure in the spinal cord dorsal horn, which sensitizes the somatosensory pathway. Glutamate and its N-methyl-D-aspartate receptor are highlighted as central actors in the complex process of central sensitization. Moreover, loss of inhibitory activity in the brain following amputation causes a disruption of the interplay between excitatory and inhibitory input [13]. In PLP, low threshold afferents might become functionally integrated with ascending spinal projection neurons. Peripheral nerve injury causes degeneration of C-fiber terminals in lamina II, a process followed by sprouting of A-fiber terminals into atypical regions of the spinal cord. Consequently, A-fiber signals might be interpreted as noxious stimuli to provoke allodynia. Thus, theoretically, PLP may occur if A-fibers invade areas of the spinal cord that normally represent the deafferented amputated limb [1]. The attributed shared role of central sensitization in the pathogenesis of both PLP and fibromyalgia is a recurrent theme in contemporary research, an acknowledgement that these two phenomena might involve a shared mechanism. Nevertheless, Brazenor et al. (2022) [14] in a comprehensive literature review found no convincing evidence showing that central sensitization can persist as an autonomous pain generator after the initiating injury has healed, and Velasco et al. (2024) [15] in their recent review of the literature found no evidence demonstrating central sensitization in humans.

The cortical remapping theory (CRT): CRT tries to explain the neurophysiological basis of PLP by suggesting that the brain responds to limb loss by reorganizing its somatosensory maps. This means, for example, that neurons previously receiving input from the arm prior to amputation become respondent to sensory input from the face, thus allowing for an experience of pain in a non-existent part of the arm when the face is stimulated. Such central reorganization of neuronal networks in the cortex may or may not be promoted by peripheral input from a neuroma or loss of C-fiber input [13]. Loss of gamma-aminobutyric acid-mediated inhibition could further lead to cortical hyperexcitability and somatosensory or motor map rearrangement. Notwithstanding, some rigorous investigations have found no clear relationship between cortical rearrangement and PLP [16]. Studies have found that during phantom hand movements, upper limb amputees engage sensory and motor cortical regions corresponding to those activated during movements of the intact limb [17,18]. Studies also indicate that PLP correlates strongly with intact, rather than altered, functional and structural representations of the missing limb in the sensorimotor cortex [17,19]. These findings do not necessarily refute CRT but merely highlight the fact that no theory so far has been able to give a comprehensive exposition for PLP’s pathophysiology that stays in agreement with diverse field data. Several factors may play a role in maintaining phantom limb phenomena [6]. Peculiarly, under spinal anesthesia, PLP and phantom limb sensations appear in patients who have never experienced so beforehand [20].

Subcortical theories [6]: subcortical structures such as the thalamus are suggested to have a role in PLP because the thalamus can act as a sole source of pain-signals. Research has demonstrated that, following spinal cord injury, neurons of the thalamus become hyperexcitable independently of spinal input, supporting the idea that the thalamus can serve as an autonomous pain generator [6,21]. In a study using a rodent model, forelimb amputation apparently led to reorganization in the ventral posterior nucleus of the thalamus which relayed new input to the deafferented cortex [22]. These findings provide further credibility for thalamic contribution in cortical reorganization and possibly PLP [6].

Proprioceptive theories [6]: are based on the idea that the brain has proprioceptive memory of the body’s position, which in certain pathological conditions might be responsible for creating pain. Evidence in support of this idea may be seen in amputees that occasionally report feeling that their phantom limbs are stuck in the last positions they remember prior to amputation [23]. Other theories suggest that PLP may result from a dissociation between proprioception and vision in the brain and may be the basis for vision-based therapies (e.g., mirror therapy and virtual reality) [24]. Due to a lack of sensory input after deafferentation, the disturbed balance of normal cortical circuitry disinhibition may possibly unmask new mis-localized sensations following amputation, contributing to PLP.

Use-dependent brain plasticity based on long-term potentiation and resurfacing of pain memory prior to surgery are additional mechanisms claiming theoretical relevance [13].

Peripheral nervous system theories: according to research, peripheral factors alone cannot drive PLP [1,6]; rather, peripheral pathologies may work in conjunction with the CNS to cause PLP. Neuromas and the dorsal root ganglion (DRG) may be essential contributors for PLP [1]. During limb amputation, inflammation and sprouting occur as DRG axons are disconnected from their distal targets where a neuroma may be formed. The injured axons and remaining nervous tissue may generate ectopic spontaneous activity from hyperexcitable loci, transmitting afferent neural signals from the periphery to the spinal cord and brain [6]. Tapping a stump neuroma can trigger pain in the phantom limb as well as the stump [13]. Damaged and reorganized peripheral nerve endings are another potential explanation for PLP. Sympathetic sprouting into the DRGs and sensory-sympathetic coupling have been described in animal studies of nerve injury [13]. Still, it is not clear cut that peripheral neuronal tissue is part of the development of PLP [1].

Psychological theories: it has been put forward that PLP is, literally, the psychosomatic expression of grief over the lost limb. Despite this compelling idea empirical studies on psychological characteristics of PLP patients indicate that they tend to have normal psychological profiles [1,25]. Some of the literature in behavioral sciences presents an inaccurate picture of amputees who have PLP because many studies on PLP gather data from amputees that requested treatment for phantom pain and who were referred to mental health professionals, which suggests inherent methodological flaws such as selection and recruitment biases [25]. It seems that in PLP almost any explanation will do, provided it seems to explain.

The exceptionally high prevalence of PLP among amputees indicates a close pathogenetic link between the surgical intervention and the chronic pain that ensues. Estimates of PLP incidence differ between studies and methodologies, although in one commonly cited survey of amputees ~78% of responders reported experiencing PLP [6,26]. While several theories try to explain phantom pain, more than one mechanism may be attributable to the generation and maintenance of the pain. As noted, empirical findings suggest a role for the CNS in PLP, and studies have found evidence for the role of the brain and memory; however, empirical data seem to be contradictory [6]. Regardless, current theory-based treatments are suboptimal (or disappointing), making PLP a ruthless disorder for patients. Interventions such as mirror therapy and targeted neuroma interventions have shown partial success for certain patients in part of the studies, indicating the value of these approaches despite the ongoing challenges and suboptimal overall treatment landscape. On the other hand, several studies, including large surveys of patients with amputations, have shown that most treatments for PLP are ineffective and do not take account of the mechanisms underlying the production of the pain [1]. The following section aims to offer an alternative mechanical mechanism for intractable postamputation spontaneous sensations and PLP as a clinical manifestation of a neuro-biomechanical disorder of the (fascio)musculoskeletal biotensegrity system, brought forth by amputation.

## 3. Methods

To apply the pathogenetic model previously suggested for fibromyalgia for PLP, a literature review was conducted on phantom limb pain and myofascial tissue in MEDLINE and Web of Science searching phrases on phantom pain, myofascial tissue, fibroblast, and tensegrity. All searches were performed in all fields for all studies with no time limit. Only studies published in English language were included in the review. Papers published in unranked journals according to the journal citation reports (JCR) were excluded. Additional literature was added by searching for sub-topics such as neuroma and unexplained complications of invasive interventions (searching the phrase: “unexplained”) and other related topics that are derived from the theoretical model (e.g., needling, biotensegrity, myofascial chains). A total of 174 records were included.

## 4. A Theoretical Biophysical Model to Help Explain PLP Pathogenesis

### 4.1. Building Blocks for a Model of Biophysical Tensegrity

**Figure 1:**
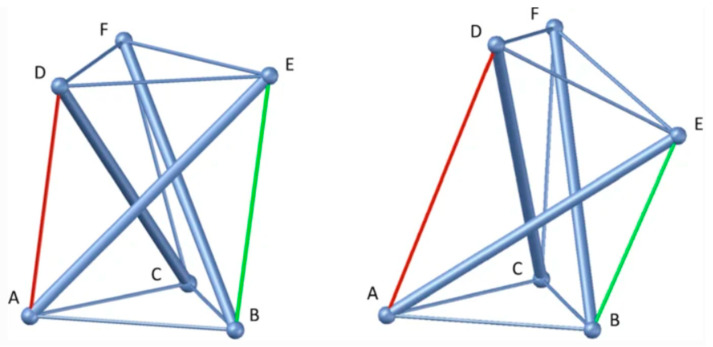
A simple free-standing three-dimensional tensegrity structure in a symmetric (**left**) and a non-symmetric (**right**) configuration. Floating compression elements transfer forces through the tension elements. The non-symmetric state occurs as the green cable is shortened while the red cable is lengthened. Changes to one node affect mechanical steady-state of the structure and other nodes as well. If a neuroma is located at, or near, a node in the system where mechanical force is transmitted to, that force could trigger pain and other sensations even when the stimulus is applied further away from the neuroma, because of tensegrity links in peripheral tissue. Mechanical forces may be chronic (e.g., sustained by tissue remodeling, tissue fibrosis, and tissue contracture) or acute (e.g., myofibroblast myosin light chain kinase mediated contractions induced by mechanical stimuli such as palpation). Figure from Micheletti A. & Podio-Guidugli, P. (2022) [29], under open access license permission.

In this section a myofascial biotensegrity-based model is presented for later explaining PLP. The proposition that fascial tissue has a key role in psychosomatic pain syndromes, which might be overlooked in PLP, stems from a theoretical model previously described in more detail elsewhere for fibromyalgia syndrome [7]. This theory describes how mechanical changes in the fascio-musculoskeletal system can help explain idiopathic chronic widespread musculoskeletal pain and fibromyalgia-type psychosomatic symptoms [7]. The model is concisely outlined as follows on one leg based on five fundamental points:

i. Tensegrity: The extracellular matrix and fascial tissue make up a complex dynamic multifunctional three-dimensional interconnected network of connective tissue that extends throughout the human body, surrounding, permeating, and connecting muscles, epimysia, perimysia, tendons, ligaments, retinacula, septa, aponeuroses, blood vessels, epineuria, periostea, and connective tissue sheaths at various depths and layers, while undergoing constant remodeling and exhibiting tensegrity-type properties [7,27,28]. ‘Tensegrity’ is an architectural term basically referring to the interplay of compressive and tensional element forces that enable the dynamic behavior and stabilization of one connected structure [27,29]. The concept of ‘biotensegrity’ integrates complex biological aspects of living systems into a biophysical model where each “separate part” of the system is valued with relation to the whole [30]. Biotensegrity provides a more practical and synergistic view of the human body as a functional system requiring both movement and stability. It upgrades the century’s-old over-simplified concept that the skeleton is the frame upon which soft tissue is “draped,” and instead, implements more complex biomechanical concepts, more fitting of real living vertebrates [28,30]. Figure 1 below shows a simple tensegrity structure as an illustration. The displayed structure is stabilized by compression and tension force elements (e.g., bones and muscular-tendinous-fascial tissue in our context). Alterations in one part of the system affect other force elements and the overall state of the entire system as well. Box 1 summarizes the molecular and cellular composition of fascia.

Box 1 **Molecular and cellular composition of fascia.**

Fascia is an integral part of the fascia-musculo-skeletal system. At a microscopic and molecular level, the fascia is primarily composed of the ECM, which provides its structural integrity and mechanical properties [28]. Key molecular and macromolecular components of the ECM include (i) collagen fibers: the most abundant protein in fascia, primarily Type I and Type III collagen, providing tensile strength and stiffness. Collagen crosslinking affects the properties of the material. (ii) Elastic fibers (elastin and fibrillin): while less abundant than collagen, elastin provides elasticity and resilience, allowing the tissue to stretch and recoil. (iii) Proteoglycans, glycoproteins, and Glycosaminoglycans: these highly hydrated molecules (e.g., hyaluronan/hyaluronic acid, chondroitin sulfate, dermatan sulfate) form a gel-like substance that fills the space between collagen and elastin fibers. They contribute to the viscoelastic properties of fascia, allowing for tissue lubrication, hydration, and resistance to compression. The sulfate and carboxyl groups on many glycosaminoglycan sugars impart a strong negative charge to the molecules. Hyaluronan is abundant in loose connective tissue and has no sulfate groups. (iv) Fibronectin and Laminins are adhesive glycoproteins that help link cells to the ECM and organize the matrix components [28]. (v) Matrix metalloproteinases are a family of zinc-dependent endopeptidases that play a crucial role in the dynamic processes of extracellular matrix (ECM) remodeling, both in physiological and pathological conditions. In the context of fascia, matrix metalloproteinases are vital for maintaining tissue homeostasis, mediating degradation, and facilitating the turnover of ECM macromolecules. (vi) Water. The negative charge of glycosaminoglycans attracts water forming a hydrated gel. (vii) Ions. pH affects the properties of fascia.

**Figure 2:**
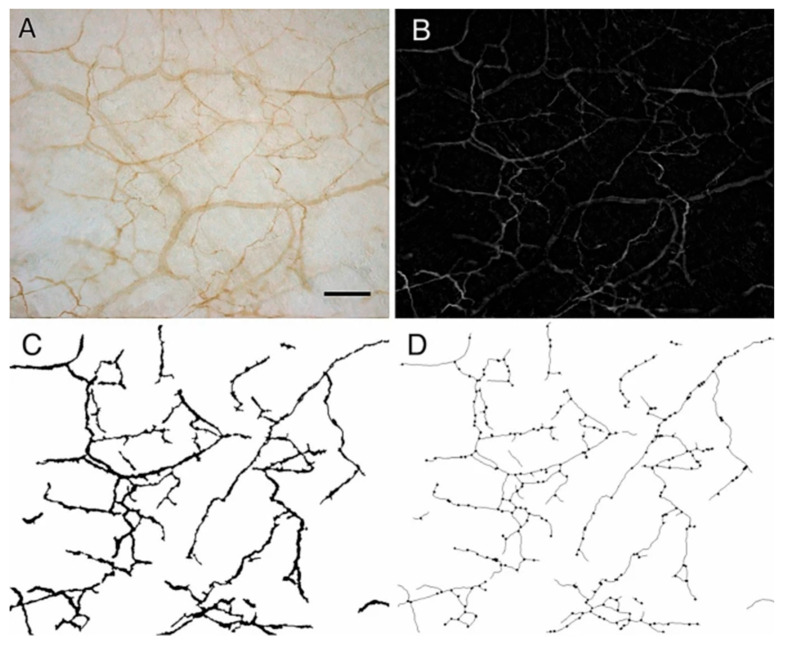
Image from open access publication by Fede & Stecco et al. (2021) [38]. Images after processing and analysis of nerve fibers in sampled fascia from mice. Scale bar: 100 µm. (**A**) original image of S100 reaction; (**B**) top-hat filter applied to enhance the contrast of picture (**A**); (**C**) binary image of nerve network; (**D**) after final analysis, binary skeleton derived from picture (**C**), with branching points marked on image.

In terms of cells, the normal cellular composition of fascia consists primarily of:

- Fibroblast cells: fibroblast are a diverse family of cells that are crucial for synthesizing and regulating the ECM. Myofibroblasts, a phenotype that has smooth muscle cell-like behavior, can also be found.
- Adipocytes: fat cells can be found within fascial layers, particularly in the superficial fascia.
- Various immune cells, such as macrophages, might be present, especially in inflammatory conditions.
- Mast cells: these immune cells are found in connective tissue and can release inflammatory mediators.
- Vascular cells: endothelial cells line the blood vessels that course through the fascia.
- Nerve cells: sensory nerve endings, nociceptors, mechanoreceptors, proprioceptors, and sympathetic nerve fibers are embedded within the fascial network. While technically neural tissue, they are inextricably linked to the surrounding ECM and interstitium.

ii. Soft tissue kinetic chains are load-bearing myofascial pathways: The (fascio)musculoskeletal system is capable of transmitting mechanical forces to a distance by means of myofascial chains [31]. Myofascial chains are anatomical mechanical links that exist in the human body and allow for force transmission to nearby and distant body regions via continuity of muscular-tendinous-fascial tissue. In this way, for instance, force in the lower limb (e.g., hamstrings) can cross joints and be transmitted to the trunk and affect the lumbar musculature (e.g., via sacrotuberous ligament and thoracolumbar fascia) [32].
iii. Mechanical and chemical alterations (densification, fibrosis, pro-inflammatory substances, shear strain, etc.) within the myofascial system can lead to the development of pain [33,34,35], and may help explain myofascial pain syndrome [28,33,36] and fibromyalgia syndrome [7]. Fascia contains a dense network of sensory nerve endings and nociceptors that play a part in the perception of pain [35,37]. In Figure 2A–D below a magnified sample of the innervated fascia is seen, with an impressive network of sympathetic nerve fibers as was demonstrated by Fede et al. (2021) [38]. Myofascial tissue (superficial fascia, perimysium, endomysium, etc.) is richly innervated and contains mechanoreceptors, proprioceptors, and nociceptors, thus playing a role in the generation of pain [28,39]. Abnormal mechanical forces and nociceptive inflammatory mediators secreted by myofibroblasts and local cells (e.g., tumor necrosis factor-alpha, interleukin 1-beta, substance P and neuropeptide Y) may trigger pain via activation of local peripheral sensory receptors. When peripheral nociception is activated, neuronal signaling is then relayed to the nervous system through spinal nociceptors that project to the thalamus and then onward to cortical and subcortical brain network areas that are responsible for pain. Also, extracellular matrix (ECM) stiffness seems to be a crucial factor in the behavior and function of nerve cells [40]. Researchers have investigated the effect of substrate matrix rigidity on neuronal cells in vitro, and found a marked difference in growth dynamics, synaptic density and electrophysiological activity of cortical neuronal networks when comparing cultures grown in substrates with 100-fold differences in Young’s modulus [41]. Matrix stiffness is a significant parameter that modulates Schwann cell function and behavior [42]. Box 2 describes biomolecular empirics that are at the basis of the matrix–neuron interactions in this model.

Box 2 **Effects of ECM stiffness on neuronal cells as a main element of the fascial theory.**

A 2016 study investigated the role of matrix rigidity on the formation and activity of cortical neuronal networks in vitro [41]. Stiff substrates of ~500 kPa in Young’s modulus were made in polydimethylsiloxane, the Young’s moduli of the elastomers and hydroxy-PAAm hydrogels were measured by dynamic mechanical analysis. The study found that a similar density of cortical neurons (50,000 cells/cm^2^) was deposited on soft micropatterned substrates (5.0 ± 0.3 kPa) and observed with differential interference contrast and fluorescent microscopy. As observed on stiff substrates, cortical neurons were homogeneously distributed at 6 h after seeding, and started to accumulate in circular islands from day 4 onwards. Meanwhile, actin was concentrated in circular islands and ßIII-tubulin was found both in linear tracks and circular islands. In contrast to stiff substrates, cortical neurons on the grid micropattern maintained their topological confinement in vitro for only 13 days. Cortical neuronal networks formed on soft substrates showed the formation of thick neurite bundles with accumulated tension that often lead to the formation of topological defects after day 13. Decreasing matrix stiffness enhanced the motility of cortical neurons. To investigate intrinsic neuronal excitability, researchers employed whole-cell patch clamp experiments on neuronal networks grown on soft (*n* = 27) and stiff (*n* = 31) surfaces from eight different cultures. This involved injecting a 500 ms hyperpolarizing current pulse of −40 pA, followed by a 500 ms depolarizing step current pulse ranging from 0 to 200 pA in 10 pA increments. Whole-cell patch clamp experiments were performed on *n* = 27 (soft surfaces) and *n* = 31 (stiff surfaces) neuronal networks using a total of 8 different cultures. The number of action potentials was counted up on each depolarizing step. Results showed that the mean maximal number of action potentials was lower on soft matrices (*n* = 4 ± 3) than on stiff matrices (*n* = 12 ± 4), suggesting that the intrinsic excitability of cortical neurons is enhanced on stiff matrices Membrane potential threshold for triggering the first action potential was similar on soft (−31 ± 1 mV) and stiff (−30 ± 6 mV) surfaces. Interestingly, the authors only observed the presence of miniature synaptic currents on stiff surfaces, confirming that matrix stiffness modulates the electrophysiological activity of cortical neurons. The findings from this study demonstrates that cortical neurons can not only sense mechanical cues from their microenvironment, but also integrate these parameters into synapse connectivity and electrophysiological activity.

A 2012 study [42] investigated the influence of substrate stiffness on the behavior and functions of Schwann cells in culture. They prepared polyacrylamide gel substrates with varying Young’s elastic moduli (4.42, 7.45, 9.10, and 12.04 kPa). Rat Schwann cells were then cultured on these substrates for various durations. Using assays like crystal violet staining, MTT, EdU labeling, time-lapse video, immunocytochemistry, RT-PCR, ELISA, and Western blot, the study assessed cell adhesion, survival, proliferation, migration, cytoskeleton arrangement, neurotrophic factor (ciliary neurotrophic factor, nerve growth factor, brain-derived neurotropic factor) expression and release, and adhesion-related protein (N-cadherin, β-catenin) expression. For visualization of actin cytoskeleton, cell samples were observed under a confocal laser scanning microscope. The findings demonstrated that the 7.45 kPa substrate provided an optimal microenvironment for Schwann cells, significantly enhancing their adhesion, spreading, survival, proliferation, and migration, as well as promoting higher expression and release of neurotrophic factors and increased levels of N-cadherin and β-catenin as shown in Western blot analysis [42]. MTT assay showed that after 24 h culture, the cell viability of Schwann cells grown on the 7.45 kPa substrate was significantly higher than that on each of other 3 substrates. The study further analyzed the distribution of Schwann cells in different phases of the cell cycle, and found that the percentage of cells in S-phase was 5.10, 37.31, 18.30, and 11.29% for Schwann cells on the 4.42, 7.45, 9.10, and 12.04 kPa substrates, respectively. FITC-labeled phalloidin staining revealed that cell attachment and spreading were more evident for Schwann cells grown on the 7.45 kPa substrate, as featured by the intensity of the fluorescence signals of actin, and this cytoskeletal arrangement was markedly different from that for Schwann cells grown on the 4.42 or 12.04 kPa substrate. Quantitative real-time PCR showed that the mRNA level of (ciliary neurotrophic factor, nerve growth factor, or brain-derived neurotropic factor was significantly higher in Schwann cells grown on the 7.45 kPa substrate than in Schwann cells grown on other 3 substrates; In line with this, an ELISA assay was used to determine the protein level of ciliary neurotrophic factor, nerve growth factor, or brain-derived neurotropic factor released by Schwann cells, and found that after culture for 3, 5, and 7 days Schwann cells on the 7.45 kPa substrate could release the higher level of ciliary neurotrophic factor, nerve growth factor, or brain-derived neurotropic factor than Schwann cells on other 3 substrates but significant difference appeared only for nerve growth factor comparisons [42].

**Figure 3:**
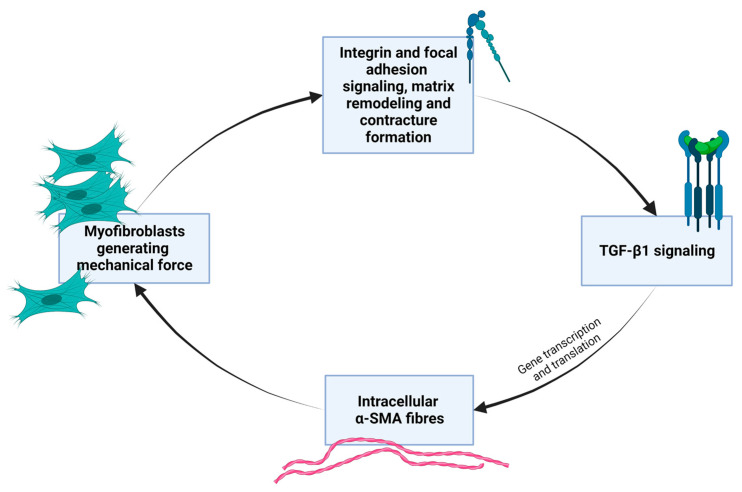
The positive feedback loop of myofibroblasts as the basis for the mechanism of this pain entity. This process, when occurring in myofascial tissue resident myofibroblasts, was suggested to be the basic cellular mechanism to drive “primary fibromyalgia syndrome” (7). TGF—transforming growth factor. SMA—smooth muscle actin.

**Figure 4:**
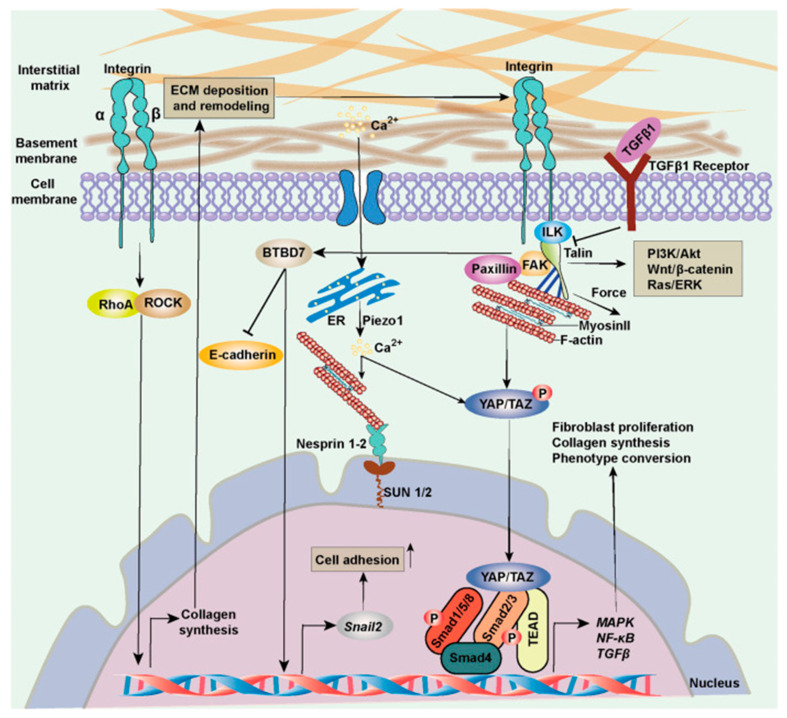
Cellular mechanotransduction and ECM stiffness. Fibroblasts are sensitive to the ECM’s composition and properties, and they deposit material in response to signaling cues. Integrins and transforming growth factor beta are central to fibroblast cellular activity. When biomechanical signals are sensed by integrins, this is transduced into cells. p38MAPK signaling, nitric oxide, and reactive oxygen species are activated to trigger downstream cascades, in collaboration with rho and ERK signaling [55]. This ultimately regulates cell phenotype, alignment, and function. The nuclear membrane is also responsive to the cytoskeleton in terms of gene transcription [55]. Figure taken from open access publication of Di et al. (2023) [55]. ECM—extracellular matrix.

To better understand the influence of the mechanical environment on neurite behavior, which is crucial in the development of peripheral nerve repair solutions, a 2020 study developed collagen hydrogel substrates with controlled stiffness gradients (ranging from 1 to 10 kPa) in their study of the mechanosensitivity of NG108-15 neural cells [40]. Using 3D-printed molds and a RAFT-stabilization process, the researchers created “Lower” (0.85 kPa mm^−1^) and “Higher” (7.96 kPa mm^−1^) gradients, ensuring a flat surface topography without altering collagen fibril structure. While neurite number and length were unaffected by absolute stiffness or gradient in the range of 1 to 10 kPa, neurite branching patterns and orientation were significantly influenced by the mechanical environment. Specifically, neurites branched more on softer segments, and their orientation varied depending on the combination of absolute stiffness and gradient slope, sometimes growing towards stiffer regions and other times towards softer regions. This research highlights that neural cells integrate both absolute stiffness and gradient steepness in guiding neurite behavior [40].

iv. Myofibroblasts express a smooth muscle cell-like behavior and are induced by mechanical strain and biochemical cues that can stimulate the process of fibroblast-to-myofibroblast trans-differentiation and proliferation [43]. The natural mechanobiology of myofibroblasts is relevant in times of scarring and wound healing in granulation tissue, but they are also found in other tissues. Myofibroblasts are cells normally found in fascia and maintain basal mechanical tissue tone [28,36]. By synthesizing contractile protein machinery and actively remodeling the surrounding matrix material and as they are sensitive to mechanical stimuli and operate with mechano-transducing signaling pathways, myofibroblasts can generate tissue contracture in a positive-feedback loop [43]. Box 3 gives further molecular elaboration on this mechanobiological festivity. Figure 3 outlines the basic self-perpetuating loop of myofibroblast contractile activity that transpires as transforming growth factor beta both enhances mechanical contractile activity of myofibroblasts while its levels are also sustained by mechanical stress. Figure 4 shows, in general, the cellular signaling pathway concerned here, which is activated in fibroblasts in response to increased ECM stiffness and mechanical and biochemical cues.

Box 3 **A brief overview of myofibroblast mechano-activity and myofibroblast-matrix interactions.**

Even though most tissues exist under mechanical tension, their resident cells do not necessarily experience the same forces. Cells can become protected from external loads in the matrix thanks to the mechanical properties of the ECM in which they are embedded. In engineering terms, this phenomenon is termed ‘stress-shielding’, which occurs due to the matrix material that cells deposit and remodel [43]. Fibroblasts are a main cell type synthesizing and regulating the ECM. The pericellular matrix is a dynamic environment that profoundly affects cell behavior. Many of the key cell–cell and cell–matrix interactions that eventually regulate fibrosis are mediated by different types of integrins [44]. When exposed to mechanical tension and various signaling stimuli, fibroblasts undergo differentiation into proto-myofibroblasts. The proto/myofibroblast phenotype develop actin stress fibers within the cytoplasm that anchor at fibronexus adhesion sites [43].

Focal adhesion complexes, intricate macromolecular assemblies comprising integrins, talin, paxillin, and focal adhesion kinase, connect the internal cytoskeleton to the ECM, enabling proto/myofibroblasts to exert forces of traction and transmit contractile tensions onto the ECM. Over time, these forces strengthen and stabilize the matrix through the deposition of new ECM material and ongoing matrix remodeling [43], specifically by depositing and cross-linking collagen I and III, fibronectin, hyaluronan, and proteoglycans. The yes-associated protein (YAP) transcription regulator pathway regulates myofibroblast differentiation and ECM remodeling in response to mechanical strain [45]. Besides the secretion of various cytokines and growth factors, the myofibroblast phenotype has increased expression of ED-A fibronectin and alpha-smooth muscle actin (α-SMA), the latter which itself is directly correlated with increased force generation by the cells [43]. α-SMA-mediated force generation fuels a positive feedback loop for myofibroblasts within granulation tissue and is a basis for a vicious cycle. The cycle transpires as tissue tension facilitates transforming growth factor beta 1 (TGF-β1) signaling, a pathway which induces α-SMA expression—thus allowing for stronger contractions. The α-SMA promoter contains a TGF-β response element [43]. Subsequently, as cells contract with additional force, more mechanical tension is sensed, which again enables TGF-β induction and then, in turn, more α-SMA expression repeatedly. In this way, myofibroblasts generate the mechanical conditions that support and enhance their contractile activity in a detrimental cycle [43]. TGF-β is secreted in a latent form to the ECM and converted to its active form via cleavage of its latency associated peptide [43].

Proto-myofibroblasts, which are a variation in the regular tissue-resident fibroblasts, arise in response to injury or pathological stimuli [43]. In normal tissues, proto-myofibroblasts are always present where there is the need to generate mechanical tension, and their formation requires platelet-derived growth factor [43]. The proto-myofibroblast is a precursor to the fully differentiated myofibroblast and can also migrate and orient themselves in cohort. A molecular feature is the rearrangement of cortical, membrane-associated actin into cytoplasmic filamentous actin stress fibers. Crucially, at this stage, these stress fibers are generally α-SMA-negative. This is considered to be a main attribute that distinguishes them from mature myofibroblasts. The transition to the mature myofibroblast is marked by the de novo incorporation and expression of α-SMA into the existing stress fibers, leading to even stronger contractile activity and the formation of mature focal adhesion complexes [43].

The myofibroblast actively remodels the ECM by secreting matrix metalloproteinases and their inhibitors (tissue inhibitors of metalloproteinases), altering the structural and mechanical properties of the matrix in a context-specific manner. This enzymatic remodeling creates a heterogeneous and evolving ECM architecture, which in turn modulates cellular behavior via changes in topography, stiffness, and ligand availability. This dynamic reciprocity, when disrupted in pathological conditions, can lead to a self-perpetuating fibrotic cycle, where increased matrix stiffness sustains myo/fibroblast activation and matrix deposition. Integrins such as αvβ3, α5β1, and αvβ6 play central roles in these interactions, mediating both anchorage to ECM ligands (e.g., fibronectin and vitronectin) and transducing signals to the nucleus via focal adhesion kinase, RhoA/Rho-associated protein kinase, and YAP/TAZ pathways. Notably, ECM composition itself influences integrin expression and clustering, creating spatially resolved signaling domains that regulate gene expression patterns related to contractility, survival, and cytokine secretion.

Myofibroblast contractility is mediated by the rho-kinase system and the myosin light chain kinase system. Rho signaling facilitates the nuclear translocation of yes-associated protein (YAP)/TAZ, mediating the expression of genes such as connective tissue growth factor, PAI-1, and COL1. Connective tissue stiffening promotes fibrosis by regulating integrin-mediated activation of latent TGF-β1. The mechanosensitive factor YAP-1, along with integrin beta-1 and its downstream pathways, plays an essential role in the regulation and promotion of fibrotic activity [46]. Once achieved, the tissue contracture induced does not necessarily require a continuing expenditure of energy by resident cells. Mechanical tension in pathological contractures results from cell contraction with concomitant ECM remodeling that alters the properties of the tissue. This way, myofibroblasts can transmit considerably high forces [43]. Tryptophan and serotonin seem also to be utilized as part of the normal function of myofibroblasts biology and feedback regulations [47]. Myofibroblasts generally have similar activity irrespective of their anatomical location [48], although the “fibroblast” cell type is a diverse family of cells [49]. Based on single-cell RNA sequencing for analyzing the heterogenous fibroblast landscape of mesenchymal cell subpopulations of mouse lung, Xie et al. (2019) identified and classified mesenchymal cell subpopulations, providing insight into the roles of fibroblasts in fibrotic diseases [49]. More than one protein conformations of α-SMA exist, and not all myofibroblasts express α-SMA [43] so using it as a marker for fibrogenic cell activity in skeletal muscle or other tissues may be problematic [49,50].

v. In addition to matrix remodeling and generating pre-stress in tissue, myofibroblasts can electrically couple to nearby cells by use of gap junctions, and contract in collaboration [36,43]. Fibroblasts form a widespread reticular network of cells in soft tissue with potentially major physiological importance. Langevin et al. [51] have shown, using confocal microscopy, histochemistry, immunohistochemistry and electron microscopy, that cultured fibroblasts of mouse subcutaneous tissue as well as cultured human fibroblasts form abundant cell processes and many points of cell-to-cell contact with each other. About 30% of such processes could be followed continuously from one cell to another using confocal microscopy. Other investigators have reported data consistent with this when investigating human fibroblasts and in vivo samples [52,53]. When fibroblasts experience mechanical stimuli they initiate cellular responses ranging from cytosolic intracellular calcium concentration and adenosine triphosphate release, to activation of intracellular signaling pathway, actin polymerization, and gene expression. It is possible that oscillations of calcium waves are a main facilitator of intercellular communication of fibroblasts by fluctuations in the levels of cytosolic calcium and its effect on downstream cell signaling pathways [51]. The nature of these oscillations likely depends, among several different factors, on substrate rigidity [54].

**Figure 5:**
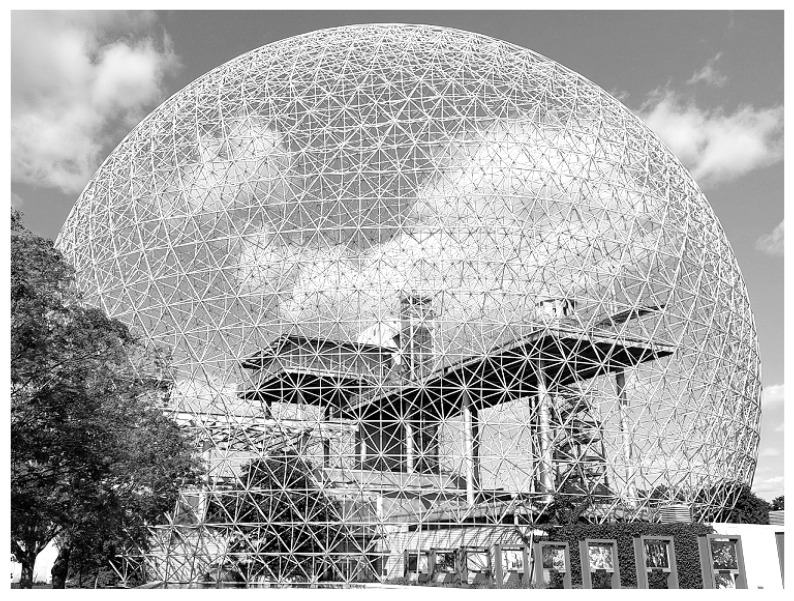
A geodesic dome as an allegory for the biotensegrity model. This is shown to simplify the idea of interrelatedness of myofascial tissue and mechanical connections in the fascio-musculoskeletal system which can enable force transmission through mechanical links and myofascial chains. In the actual model, which is dynamic and in movement, each segment can have a different spring constant that changes according to the ECM composition and cellular activity of cells and the state of the fascia, the temperature, hydration, and so forth. In a biotensegrity framework, any shift or imbalance in the biotensegrity system may potentially lead to clinical manifestations, depending on the area, depth, force, and anatomical structures involved [7]. Amputation in one part of the system causes disruption in the stability of the entire system and the structures within it. After a disruption, caused by surgery, the structure has imbalance in it which may cause symptoms such as pain when severe enough to alter physiological processes. If a neuroma is present in a location experiencing high tensional force, it may predispose to pain and hyperexcitability of its sensory pathway.

The above findings are the basis for the myofascial biotensegrity-based model. In essence, this mechanism can be summarized as: myofibroblast-mediated tensegrity tension, ECM rigidity, and compression, with subsequent corresponding neurophysiological aberrations, on a background of interrelated connective tissue and myofascial chains. It is essentially a mechanobioneurological disorder and a myofibroblast-driven disease of the fascio-musculoskeletal biotensegrity-like system, whose severe manifestations would be analogous to a sort of mild-to-moderate global chronic exertional compartment-like syndrome. A more in-depth analysis of this theoretical model in the context of fibromyalgia can be found in a recent study [7]. To illustrate the model, Figure 5 depicts a dome structure as a simplification of this framework, representing the human body and the mechanical forces that travel within the system via mechanical links. Tension in fascia in one area of the system allows for the transmission of mechanical forces throughout the connected nodes of the structure. The epimysial fascia, for example, is capable of transmitting forces between adjacent synergistic muscular fiber bundles both belonging and not belonging to the same motor unit [33]. It is suggested that there is an anatomical continuity between epimysium and paraneural sheath, so the anatomical structures they surround may be sensitive to unbalanced tension in the epimysial fasciae. Such imbalance and tensile forces have a potential of impacting the paraneural sheath, thus limiting displacements of nerves and contributing to noticeable clinical manifestations [56]. In general, disease occurs, under a biotensegrity framework, when unbalanced tensegral forces and mechanical compression are sufficient to alter physiological processes and manifest clinically.

### 4.2. Examples of Post Intervention Complications and Pain: Exploring a Biophysical Framework of Tensegrity

In this section, empirical evidence is reviewed to explore the applicability of the proposed biotensegrity model as part of our theoretical discussion. The main motif in analyzing the following cases under this framework is that interventions involving fascial tissue alter pre-stressed tensegrity dynamics in the extracellular matrix, potentially leading to pain and related symptoms. The complications below might be suspected to result from abnormal mechanical forces within myofascial tissue and interstitium, which can be detected by sensory receptors.

- Plantar fasciitis—A study involving 37 patients with plantar fasciitis treated with corticosteroid injections at the calcaneal origin reported that 30% experienced a sudden tearing sensation in the heel, while others exhibited gradual symptom progression [57]. While the injections alleviated initial heel pain, new complications soon emerged, including metatarsal pain, midfoot discomfort (dorsal and lateral), foot weakness, swelling, and metatarsal fractures. All cases showed evidence of plantar fascia rupture [57]. The new symptoms eventually resolved in most patients within a period of one year but for others the symptoms persisted.

Another study encountered the same conundrum. While several patients with plantar fasciitis experienced a sudden rupture associated with a corticosteroid injection, others had a gradual onset of symptoms. Despite relief from the original pain, complications such as lateral plantar nerve dysfunction, longitudinal arch strain, midfoot strain, hammertoe deformity, and stress fractures were documented. A diminished tension of the plantar fascia was consistently demonstrated in affected patients via stretch tests [58].

According to the biotensegrity model, these post-injection complications may not simply be adverse effects of injection, but direct results, because the treatment modality changed the biotensegrity system by acting as a mechanical intervention of the fascia. In this framework, complications occurred due to the release of forces in fascia in areas of very high tensional force with a sudden change in the pre-stressed tensegrity’s state [7]. A sudden mechanical alteration can shift existing forces to other areas and exacerbate imbalances. The mechanism by which complications resolved over time is expected to happen via tissue regeneration and healing, and ECM remodeling which stress-shields the area over a period of time. Moreover, steroid injection is shown to decrease fibromatosis and myofibroblasts in adhesive capsulitis [59], suggesting that injection to the plantar fascia may alter myofibroblast generated tensegrity forces [7]. It is possible that tension rather than entrapment causes the nerve dysfunction following plantar fascia rupture [58]. Clearly, post-injection complications (e.g., metatarsal pain, nerve dysfunction) may not be solely the consequences of biotensegrity disruption, and other potential mechanisms directly related to steroid injection may be involved (e.g., tissue weakening, altered biomechanics, direct neurotoxicity, etc.). However, a difficulty arises in the next examples, since any explanation that relies on the effects of steroids, and any non-tensegrity explanation, leaves other anomalies unsolved, as discussed in reported phenomena such as:

- New onset Boutonniere deformity develops after treating Dupuytren’s disease [60].
- Trigger finger is more likely to occur following carpal tunnel release [61].
- Treatment for lateral epicondylitis requires “unrelated” arthroscopic decompression of the shoulder joint [62].
- Compartment syndrome of the foot occurred following spine surgery [63].
- Needling and/or injection to the contralateral limb may relieve PLP [64,65].

These findings are not surprising when recollecting the principles of biotensegrity, because fascia functions as a continuous myofascioskeletal network with a complex biomechanical behavior. Connective tissue directly links most skeletal muscles in the human body [31,66], and fascia is continuous from the trunk across the upper and lower limbs [67]. Acute bout of stretching of the upper limbs increases maximal range of motion of the distant lower limbs and vice versa [68]. Isometric plantar flexion produces measurable changes in the stiffness of lumbar soft tissue and gastrocnemius, demonstrated by shear wave elastography measurements; this relationship is explained by it being a part of the myofascial tensegrity system [69]. Unilateral stretching of one leg increases range of motion of the contralateral leg, which is explained by the involvement of continuous structures such as the myofascias and the peripheral nervous system that form a link between the lower limb and the spine [67]. The neck, head, and eyes are arguably linked by anatomical myofascial continuum [70]. Muscle and fascial tissues do not exist in isolation, but rather they function together to facilitate the body’s movements through a mutual connection forming a myofascial tensional network to connect all parts of the body as a whole [69].

The studies cited above describe phenomena that are anomalies (anomalies defined here as phenomena that were not expected). Our discussion here focuses on the theory. It is true that the pathophysiological link drawn between these phenomena and tensegrity disruption is highly speculative and not explored so far, therefore there is no literature on it, which calls for empirical studies to test it. The aim of this discussion is to discuss a mechanism for the anomaly. Obviously, since these are anomalies, there is not much convincing evidence supporting any known mechanism so far. Hence, any pathophysiological connection remains speculative at this point. The discussion has not claimed that tensegrity is the state of the art in PLP or plantar fascia rupture complications. It merely acknowledges that biotensegrity can predict these anomalies, and might help explain them. As with any other model, the tensegrity model described here is a simplification of reality. The purpose of a theoretical model is to simplify, explain, and predict. There is evidence for fascial functional and structural continuity between the foot, leg and back [31,32,66,68,69]. In addition, it was found that when plantar fascia rupture occurs, other anatomical and physiological anomalies occur [57]. When these two evidences are combined one can infer the presence of tensegrity type dynamics. It is worth noting that the myofascial biotensegrity-based model does not make it mandatory for the whole body to be involved in a specific situation. Because tensegrities are composed of discrete networks of support elements, rather than a uniform medium like a chunk of metal or a rubber band, they provide a way to transmit mechanical forces along specific paths and to focus or concentrate stresses on distant sites and at different size scales. These are all features observed at the level of whole organs as well as tissues, cells, membranes, cytoskeletal networks, subcellular organelles, nuclei, mitotic spindles, transport vesicles, viruses, and proteins [71]. Of course—biology is more complicated than that. Box 4 below summarizes the main points of the model. Tensegrity is not the only proposed explanation. It is one explanation that is based on this mechanical principle of tensegrity, which is the focus of the discussion. It does not exclude other explanations, but sheds light on a tissue system that is so far overlooked.

Box 4 **Recapitulating the main points for the biotensegrity-based model.**

While the pathophysiological mechanisms of persistent post-operative pain and fibromyalgia remain unclear, it is accepted that peripheral tissue and persistent nociceptive inputs have a crucial role in these chronic pain conditions and may drive them (Flor et al., 2006; Katz et al., 2021) [8,13]. The biophysical concept of biotensegrity provides a comprehensive view of the fascio-musculoskeletal system where mechanical forces act not in isolation but affect the system as a whole. In this framework changes in one part of the system—for example, due to invasive procedures—may affect other parts of the system and potentially have long-lasting clinical implications (Figure 1 demonstrates the concept of tensegrity). Interstitial proto/myofibroblasts are a main building block of the theoretical model, because they disrupt the delicate balance of the biotensegrity system by increasing tension and stiffness. The yes-associated protein (YAP) and transcriptional co-activator with PDZ-binding motif, key transcriptional regulators, are highly sensitive to mechanical strain and ECM stiffness. Increased ECM stiffness promotes their nuclear translocation and activation, leading to the transcription of genes involved in myofibroblast differentiation, collagen synthesis (e.g., COL1), and ECM remodeling (e.g., connective tissue growth factor, PAI-1). This creates a positive feedback loop where increased stiffness drives further fibrotic gene expression. This mechanical alteration, along with the release of sensitizing chemicals, can directly and indirectly activate nociceptors, leading to the perception of pain and downstream pain compensatory mechanisms, which might help explain overlapping idiopathic chronic pain conditions. The neuronal cytoskeleton itself, connected to the ECM via integrins and other adhesion molecules, is sensitive to external mechanical forces. Alterations in ECM stiffness can induce long-term transcriptional and epigenetic changes in nerve cells, affecting their intracellular cytoskeletal architecture and subsequently their electrophysiological activity. The increased rigidity and tension within the fascial network can mechanically compress embedded structures, including small nerve fibers and neuromas. This sustained mechanical pressure directly activates mechanosensitive ion channels on neuronal membranes, generating aberrant nociceptive signals. Biotensegrity, and a pathological disorder of it, can help explain various phenomena, and might be relevant in chronic pain states such as refractory post-operative pain, and is at the basis of the mechanism suggested here for fibromyalgia (in short: myofibroblast-generated biotensegrity tension, compression, and ECM stiffness). This is owing to fascia being a sensitive and sophisticated tissue that houses sensory and sympathetic nerve fibers and because pathological processes and dysfunction of fascia are suggested to promote chronic pain conditions. The sections above delineated a theoretical model and mechanical mechanism to offer a possible explanation for persistent pain in medically unexplained cases. Figure 3 and Figure 4 summarize the core molecular pathway relating to the mechanism. Biotensegrity is a building block of the theoretical model. Biotensegrity suits the model because it aligns with the interconnectedness of the myofascioskeletal system. Biotensegrity offers a simplified framework for the theoretical model, while the drama of myofibroblasts is on the foreground. In the case of PLP, clearly, the tensegrity system is disrupted mechanically after amputation. Like any other model, the biotensegrity model laid out here is a simplification of reality. The purpose of a theoretical model is to simplify, explain, and predict. Of course- biology is more complicated than that.

## 5. Amputation in a Framework of Osteomyofascial Tensegrity

### 5.1. Considering the Relevance of Biotensegrity for PLP

Besides the acute effects, invasive procedures can have long-lasting effects on tissue. In scar tissue a high number of myofibroblasts can be seen [44]. These cells create and transmit mechanical force onto the surrounding ECM using myosin light chain kinase and rho kinase mediated signaling pathways while sensing and transducing mechano-signaling via focal adhesion complexes [43]. Myofibroblasts within the fascia are suggested to contribute to the pathophysiology of myofascial pain syndrome [28]. This is due to their ability to generate abnormal mechanical forces and secrete nociceptive mediators. These factors can then activate peripheral sensory receptors, thereby triggering pain [28]. Should the local microenvironment and surrounding ECM be altered by scar tissue and interstitial myofibroblasts, these changes could facilitate nociceptive activation through mechanical or chemical stimuli. This is anticipated to generate pain and spontaneous sensations in myofascial tissue, even in the absence of apparent active injury.

Cramping and squeezing-like phantom pain was found to be related to muscle tension in the residual limb as authors have shown that electromyographic representations of muscle tension in the residual limb shortly preceded changes in such sensations of PLP [1]. Being closely associated with spasms and cramping in the limb’s muscles in PLP [1] suggests that myofascial tissue might possibly be involved besides neuropathological or neuropsychiatric reasons. Therrien et al. (2021) have found a relationship between electromyography activity and PLP in a study that investigated phantom limb movement and sensations in lower limb amputees [17]. During movement of a phantom foot, electromyography amplitude in the residual vastus lateralis was positively related to patient phantom pain ratings. The study’s authors also note that neuroma-related PLP can coexist with, but function independently of, mechanisms driving abnormal residual muscle activity. The myofascial biotensegrity-based model expects that mechano-active proto/myofibroblasts in myofascial tissue and interstitium will be involved in neuroma and PLP pathophysiology. When tensegrity disruption is localized to a certain anatomical area (e.g., a limb containing a neuroma) it is expected to manifest as a sort of “local fibromyalgia”. In terms of a tensegrity spatial structure, if the paraneural sheath participates in the tensegrity steady state, deafferentation or severing a large nerve would alter mechanical forces exerted on the residual part of the nerve. The conceptual model discussed here deals with tensegrity. The model is applied to PLP. In the case of PLP, clearly, the tensegrity system is disrupted mechanically after amputation.

### 5.2. Neuroma as a Focal Sensor of the Biotensegrity System and a Source of Sensations and Pain

Neuromas may form whenever peripheral nerves are injured [5]. A neuroma consists of tangled axons that are unable to regenerate to their target, as well as fibroblasts, and other cells. Chronic neuropathic pain after surgery is often attributed to the formation of a neuroma in scar tissue. Following a mastectomy, for example, iatrogenic damage and scarring may create an environment for neuromas to form, and axons encased within these scars can lead to spontaneous pain and heightened mechanosensitivity [5]. A stump neuroma can serve as a source of ectopic discharges and afferent input to the spinal cord, driving spontaneous pain [13]. Scar tissue around a neuroma is shown to contain myofibroblasts for a prolonged period of time [72]. Besides transmitting mechanical force to the surrounding ECM, myofibroblasts remodel the ECM by degradation and deposition of collagen and matrix material [43]. A microscopy and biochemical study investigated the pathobiology of neuromas in human subjects [72]. The study found scattered myofibroblasts that appeared in samples singly or as aggregates of cells. Along a dense glycosaminoglycan matrix, myofibroblast type cells were seen to be associated with each other and the surrounding collagen. Meanwhile, samples of nerves from control subjects demonstrated normal ultrastructural components such as myelinated and unmyelinated neurofibers, characteristically surrounded by Schwann cells, but without myofibroblasts. Interestingly, myofibroblasts examined from within older neuromas had almost half their cytoplasmic area taken up by intracellular filaments. The study’s authors concluded that myofibroblasts seem to play a part in the pathobiology of human neuromas [72].

In addition to actively generating mechanical tension in the surrounding matrix [43], myofibroblasts secrete cytokines such as interleukin-1 and interleukin 6 [73], which are known to mediate chronic pain pathways in nociceptors [74,75]. Given that cells of the myofibroblast phenotype generally exhibit similar characteristics independent of the anatomical location [48], their proliferation adjacent to a neuroma would instigate significant remodeling and reshaping of the local ECM. This continuous and dynamic remodeling process creates a perpetually changing mechanical landscape for the neuroma, resulting in inconstant mechanical forces exerted on neuronal cell membranes and their cytoskeletal components. Since the remodeling of the ECM environment is an ongoing dynamic process, the neuroma will experience a changing dynamic ECM microenvironment. Within this dynamically altered ECM, mechanoreceptors and nociceptors, specialized sensory nerve endings, are intrinsically responsive to mechanical stimuli, being specifically mechanosensitive. Forces like compression, stretching, or traction can induce action potential firing in these neural cells and signal pain through mechanotransduction, a process mediated by specialized mechanosensitive ion channels and stretch receptors on the neuronal cell membrane that convert mechanical stimuli into electrical signals. Sugawara et al. (1996) [76] investigated the effects of mechanical compression and hypoxia on nerve roots and dorsal root ganglia using an in vitro model with canine lumbar sample. It was shown in their study that the dorsal root ganglia were significantly more sensitive to mechanical compression than the dorsal roots, requiring less pressure to induce firing and exhibiting longer-lasting firing. Under hypoxic conditions, the dorsal root ganglia showed spontaneous firing, and their sensitivity to mechanical stimuli significantly increased.

The role neuromas have in the pathogenesis of PLP is not completely elucidated and is under debate. However, it was shown that preemptive coaptation and collagen nerve wrapping were associated with lower pain scores, neuroma formation, and phantom symptoms, as well as higher ambulation rates in transfemoral amputees [77]. Moreover, tapping a stump neuroma can induce PLP [13]. These and more findings are highly suggestive of neuroma involvement in PLP. According to the model presented above, the mechanosensitivity of a local traumatic neuroma formed after amputation is expected to predispose to pain and seemingly arbitrary sensations whenever myofibroblast-generated ECM tension and rigidity influence the neuroma with sufficient mechanical force. Forces like compression, stretch, or traction can induce action potential firing in these neural cells and signal pain through mechanotransduction, a process mediated by specialized mechanosensitive ion channels such as Piezo1/2 and other stretch-activated receptors on the neuronal cell membrane that convert mechanical stimuli into electrical signals. The neuroma has intrinsic hyperexcitability (e.g., due to inflammatory milieu, ion channel dysregulation, ectopic electrogenesis) and is not merely a passive actor. Mechanical stimuli that are sensed by the neuroma might modulate the neuromas intrinsic behavior and its ectopic noxious afferent input transmitted to the spinal cord and brain. That the perceived sensations may manifest as more complex (e.g., in a missing part of the limb) can also be explained by pathologies downstream to this, like axonal sprouting, electrical coupling with surrounding cells via gap junctions and additional central neurobiological changes (e.g., cortical remapping) which may also potentially affect the conscious sensory experience. Having said that, PLP seems to involve both peripheral and central processes working in concert to facilitate the perception of pain and somatic sensations in the limb [1]. Simply because pain is perceived as located in a non-existing part of the body does not necessarily mean that the cause is in the brain.

Although most therapies for PLP focus on the brain, myofascial trigger points in residual limb of amputees may play a part in postamputation chronic pain. A study has found that an intervention targeting myofascial trigger points leads to marked resolution of phantom and stump pain in lower limb amputees [78]. Investigators of PLP reported finding a constant link between the localization of taut myofascial bands in the residual limb and the localization of PLP. These researchers observed typical dynamics whereby applying pressure to myofascial taut bands on the posterior aspect of the residual limb typically induced a phantom pain localized to the calf, spreading to the heel, sole, and under and between all toes. Additionally, a distinctive pattern of pain distribution was found while applying pressure to the different taut myofascial bands in the stump. Pressure applied to the bands for more than a few seconds triggered a strong local pain, as well as a centrifugally referred pain to the distal part of the stump, and also a PLP in the missing part of the limb, that was of either cramping, aching, burning, or pressing in its pain characteristic. Tip pressures to the taut myofascial bands and trigger points, when applied in the proximal part of the stump of five transtibial amputees, provoked aching or burning pain localized to the distal stump in the section below the knee joint, as well as a PLP [78].

For a reader familiar with biotensegrity, the dynamics described above may raise suspicion of underlying tensegrity biomechanics being in play, meaning that treating PLP and co-occurring stump pain by targeting nearby myofascial tissue seems totally reasonable. If myofascial tissue affects the neuroma through biotensegral mechanical ECM links, PLP will be deeply influenced by the mechanical state of both adjacent and distal myofascial tissue and the overall system’s state: both ECM substrate rigidity and overall imbalance. Substrate stiffness and rigidity influences behavior and function of nerve cells [40], including their growth dynamics, synaptic density, and electrophysiological activity [41]. It would not be unreasonable to suggest, therefore, that ECM biophysical and biochemical properties affect, either directly or indirectly, neuroma.

### 5.3. Flexion Contracture and Its Relevance to a Tensegrity Framework

Flexion contracture is a fixed abnormal flexion occurring in a joint that limits full extension and can be caused by several factors, including muscle imbalance [79,80]. During prolonged immobilization of a joint fibro-fatty connective tissue starts to invade into the joint space as early as two weeks after immobilization. Afterwards fibrous adhesions occur and further compromise the mobility of the joint. Immobility allows for the development of abnormal cross linking between connective tissue fibers [79]. Contractures of more than 25 degrees angle occur in approximately 13% of those who with transtibial amputation and 23% in those with transfemoral amputation [81]. Etiology of a fixed flexion knee deformity relates to bony impingement, ligament contracture, posterior capsular contracture, and hamstring shortening, which can all facilitate an inability to fully straighten the joint [82]. Some authors have suggested that contractures may be caused by prolonged immobilization or improper positioning because lack of movement induces adhesion of synovial membranes and promotes a contracture [79,81]. Immobilization-induced joint contractures can be promoted by two types of components: arthrogenic (relating to bone, cartilage, synovial membrane, capsule, and ligaments) and myogenic [83]. In rat models, immobilization leads to knee contracture and fibrosis of the joint capsule with an increase in the number of cells of the myofibroblastic phenotype, in a positive proportion with the length of immobilization time [83]. One study documented microscopy findings following implantation of a silicone tendon prosthesis for deep flexor tendon reconstruction [84]. An extensive, dense, contractile fibrosis soon developed, resulting in a flexion deformity that ultimately required amputation. When examined in light and electron microscopy, the amputated specimen was populated by myofibroblasts seen in the new tendon sheath, which probably caused the deformity because of their contractile properties [84]. Fibrosis caused adherence to pre-existing aponeurosis. In addition, because these cells form connections with each other and to the stroma, when they contract the whole tissue is deformed [84].

In the context of our discussion of tensegrity, contractures indicate extreme abnormal tensional forces in the pre-stressed tensegral system. Since fascial tissue is continuous (including the deep fascia, joint capsule, tendons, etc.) the presence of contracture reflects mechanical imbalance affecting the system and therefore higher risk for chronic “non-specific” pain (i.e., myofascia-related pain). While contracture exemplifies abnormal tension, it should not be attributed only to myofibroblasts. The role of muscle atrophy, neural adaptations, structural and functional alterations due to disuse, and prosthetic interface issues in amputation-related contractures must be acknowledged besides potential myo/fibroblast ECM remodeling. Non-muscular structures such as fascia are able to limit the maximal joint range of motion during stretching [67]. In contrast to skeletal muscle fibers, myofibroblasts exert a relatively long-lasting contractile activity together with their ECM remodeling and stress shielding, which maintains chronic tissue contracture [43,85]. Myofibroblasts can generate sufficient mechanical force to influence musculoskeletal dynamics [85]. Myofascial mechanical tension can be sensed by peripheral nerves and can trigger nociception, which may underly pain in certain psychosomatic pain syndromes [7,8]. Fascial tension is likely to focus more near hard and angled surfaces that have sharp force-gradients [7].

According to the framework suggested here, in the long term, ECM remodeling and myofibroblast stress shielding alters the local mechanical forces, which can alter pain severity and characteristics. A sedentary and immobile limb, particularly when pressed against a prosthetic, experiences chronic non physiological mechanical stress cues, experienced by fascial cells. Mechanical stress serves as a stimulus for the myofibroblast contractile cascade, triggering a positive feedback loop mediated by transforming growth factor beta [43]. Consequently, more tension is created in a vicious cycle involving mechanosensitive and mechano-transduction cellular signaling pathways that involve focal adhesion kinase [43]. The stump will continually be at a disadvantage from a biotensegrity perspective even if no prosthesis is used. Interestingly, children born without a limb can experience “phantom pain” [86].

Although the myofascial biotensegrity-based model predicts a role for fascial myofibroblasts in PLP, it remains at this point theoretical since no empirical study of myofibroblasts in PLP was found while searching major databases. For example, a search for “(phantom pain) AND (myofibroblast)” in MEDLINE in all fields for all studies with no time limit yields zero results. Having said that, lack of evidence (yet) is not counterevidence. The same applies to other theories out there. Brazenor et al. (2022) [14] in a comprehensive literature review found no convincing evidence showing that central sensitization can persist as an autonomous pain generator after the initiating injury has healed. Velasco et al. (2024) [15] in their recent review of the literature found no evidence demonstrating central sensitization in humans. Still, though, it seems that for some, refractory chronic pain is central sensitization until proven otherwise.

### 5.4. Summary of the Neuro-Mechanobiological Model for Rethinking PLP

In summary, while the pathophysiological mechanisms of PLP, as well as fibromyalgia, remain unclear, it is accepted that peripheral tissue and persistent nociceptive inputs have a crucial role in such chronic pain conditions and may drive them. The notion of a pathophysiological connection between fibromyalgia and PLP is not new. Contemporary research frequently highlights the shared role of central sensitization in both conditions, suggesting a common underlying mechanism. This paper proposes an alternative perspective, focusing on a peripheral mechanical mechanism. The hypotheses are derived from the conceptual model, and obviously call for empirical studies to test it. The biophysical concept of biotensegrity provides a new perspective of the fascio-musculoskeletal system where mechanical forces act not in isolation but affect the system as a whole. In this framework changes in one part of the system, for example, due to invasive procedures, exert effects on other parts of the system and potentially have long-lasting clinical implications from the standpoint of ECM. Matrix remodeling by proto/myofibroblasts would thus disrupt the delicate balance of the biotensegrity system by increasing tissue rigidity. This mechanical alteration, along with the release of sensitizing chemicals, would directly and indirectly activate nociceptors, mechanoreceptors, proprioceptors, and more, which can help explain the perception of pain in idiopathic chronic musculoskeletal pain conditions. This highlights an unexplored biomechanical approach to understanding PLP.

The presence of myofibroblasts specifically within neuromas potentially carries significant practical implications for understanding and treating neuropathic pain. Their contractile activity and ability to remodel the ECM could generate abnormal, sustained mechanical tension or compression directly upon the sensitive nerve fibers within the neuroma. This mechanical stress, alongside their paracrine secretion of cytokines, could serve as a direct, localized source of nociceptor activation and sensitization. Consequently, targeting myofibroblast activity or modifying the stiffened ECM around neuromas represents a novel therapeutic strategy for alleviating intractable neuroma pain.

In this theory, myofibroblasts within fascia are thus suggested to significantly influence the biotensegrity system and contribute to medically unexplained chronic pain and phantom limb sensations by several key ways:

1. The increased tension and stiffness caused by myofibroblast activity can directly stimulate mechanosensitive nociceptors or polymodal fibers within the myofascial tissue. These nociceptors respond to mechanical deformation and pressure, firing signals that are interpreted as pain. Mechanosensitive ion channels (e.g., Piezo1/2, TRP channels, stretch-activated channels) mediate the conversion of mechanical stimuli into electrochemical signals. Also, in certain instances, the altered mechanical environment can lower their threshold for activation, making them more easily triggered.
2. Under conditions of increased ECM stiffness, long-term transcriptional and epigenetic level adaptations in nerve cells would lead to changes in their structure and function, including intracellular cytoskeleton architecture, which can affect neuronal electrophysiology. Researchers have investigated the effect of substrate matrix rigidity on neuronal cells in vitro, and found a marked difference in growth dynamics, synaptic density and electrophysiological activity of cortical neuronal networks when comparing cultures grown in substrates with 100-fold differences in Young’s modulus [41]. The pre-synaptic density was two times higher on stiff substrates and consistently the number of action potentials and miniature synaptic currents was enhanced on stiff substrates [41].
3. Excessive collagen deposition and tissue remodeling associated with myofibroblast activity can potentially lead to the entrapment or compression of small nerve fibers within the deep or superficial fascia. Involvement of the neurovascular bundle can also add to the clinical presentation.
4. The effects of higher substrate rigidity on Schwann cells [42] add another neuropathic component to the mechanism.
5. Beyond mechanical forces, myofibroblasts can release various pro-inflammatory mediators and growth factors that can sensitize nociceptors in the surrounding tissue, lowering their threshold for activation by mechanical or chemical stimuli. This can lead to hyperalgesia and allodynia.
6. Local hypoxic conditions in muscle caused by epimysial and perimysial compression can lead to the release of algogenic substances from the affected tissue, and to the activation of chemoreceptors on nociceptors, and low-grade inflammation.
7. Myofibroblasts can communicate with neighboring cells, including other fibroblasts, myocytes, and potentially nerve cells, via gap junctions. This allows for the direct transfer of electrical and chemical signals, potentially contributing to the propagation of pain signals.
8. Changes in the mechanical properties of fascia due to myofibroblasts can affect proprioception and kinaesthesia. This altered sensory feedback might lead to compensatory movements, muscle imbalances, and increased strain on other tissues, which can indirectly contribute to pain.
9. Compression of the dorsal root ganglion (and conceivably also sympathetic chains) may lower thresholds for electrophysiological activity and can even cause them to fire signals spontaneously. Increased rigidity and compressive forces in the ECM microenvironment may constitute a sufficient endogenous stimulus to independently initiate this phenomenon, in the absence of external stimuli.
10. A similar effect as for the previous point (point 9), with regard to a stump neuroma.

Finally, it is known that fascia and the tissues of the locomotor system have several layers and subdivisions as defined in textbooks, and that the classification to layers comes more from an anatomical standpoint. In reality, the ECM and fascial connective tissue is integrated as a connected network in the human body with interrelated and intermeshed tissues that are difficult to determine where one section ends and the next one begins, while they are all in relationship with one another functionally [28,30]. Therefore, the theoretical model presented here approaches fascia stemming from this standpoint—as a connective tissue network. To explain the model, there is no need to focus on a specific layer as classified in the textbook.

## 6. Discussion

In this paper a biomechanical model for PLP and spontaneous phantom sensations was proposed, shifting focus from purely neurocentric theories to a framework of dysregulation of the fascial ECM and myofibroblast-mediated disruption of biotensegrity. Essentially, it posits that amputation disrupts the body’s tensegrity equilibrium, leading to abnormal ECM remodeling by hyperactive myofibroblast cells in fascia and interstitium. This creates sustained mechanical tension and compression on sensory nerves/neuromas embedded within the ECM, generating nociceptive signals perceived as PLP. The theory integrates concepts from connective tissue biology, mechanotransduction, and clinical observations and anomalies (e.g., plantar fasciitis complications, fibromyalgia) to support its framework, and challenges prevailing neurocentric models while highlighting understudied peripheral mechanisms. What makes this mechanism here specific to PLP rather than any other myofascial pain, is mainly that the neuroma, which can be formed as a consequence of amputation, is a sensitive apparatus that can be affected by its internal state (inflammatory milieu, intrinsic ability for ectopic discharging, etc.) and its microenvironment (e.g., ECM), and can transmit signals along the tracts that normally represent the amputated part of the limb. This can help explain not only the spontaneous intractable pain, but also the non-painful sensations. This theory anticipates that stump pain and PLP should often coexist, due to localized ECM remodeling within the same anatomical region as the neuroma. Linking fibromyalgia with PLP is not a novel idea and is not tangential. The attributed shared role of central sensitization in the pathogenesis of both PLP and fibromyalgia is a recurrent theme in contemporary scientific literature, which acknowledges that these two phenomena might involve a shared mechanism. This paper offers a peripheral mechanical mechanism. When empirical studies advance, the theory can be further articulated and enriched.

### 6.1. Biotensegrity as a Useful Framework to Help Explain Osteomyofascia Phenomena and Anomalies

Research has hitherto established a key role for a neuroma in PLP, and it was previously shown that myofibroblasts are likely to be involved in neuroma pathobiology. The conceptual framework of myofascial biotensegrity here attempts to explain functional somatic syndromes and myofascial pain syndromes as variations in one medical entity united by a shared neuro-mechanobiological mechanism [7]. In this framework, variations in the syndromes’ manifestation occur, in part, because of the involvement of different anatomical sites. The development of abnormally high pre-stress is expected to manifest as PLP, phantom limb sensations, and stump pain when concentrated more locally in the limb and is detected by sensory nerve fibers and a local neuroma. The neuroma’s intrinsic neurophysiology, which can be relevant for PLP irrespective of biotensegrity, shouldn’t be disregarded or downplayed though. If a local tissue contracture or scar is created by myofibroblasts around the neuroma, in the perineurium, and in adjacent myofascial tissue, it may be able to predispose to chronic ‘arrhythmic’ ectopic signaling from the neuroma. This effect is attributed to tensional forces in the nearby ECM and forces dispersed onto embedded nerves and their cytoskeleton. Should mechanical tension, shear and hydrostatic force, elastic stretch forces or ECM rigidity eventually surpass a critical threshold for stimulating the neuroma cells, it would activate stretch receptors and mechanosensitive ion channels on the plasma membrane. This activation would increase the neuroma’s propensity to generate action potentials and drive afferent signals along the neural tract that normally represents the amputated (and/or residual) part of the limb, and manifesting in cognition. In turn, such hyperstimulation might eventually lead to peripheral and spinal genetic, epigenetic, and structural adaptations, and to even further neurophysiological effects as postulated by neurobiology. Central neurological processes, e.g., activation of the neuroendocrine stress response, mesolimbic dopamine circuitry adaptation, endogenous opioid response, functional and structural synaptic changes in the spine and brain, microglia activation, changes in gene expression and transcriptome, post translational protein modifications, etc., are likely involved in the compensation and adaptation to this peripheral organic process.

Psychosomatic and neuropsychiatric medical conditions such as fibromyalgia are often thought of as having no peripheral organic finding to explain them, although this is in fact not necessarily true. Crucial evidence suggesting that connective tissue and musculoskeletal pathologies may underly fibromyalgia symptoms can be found in various studies [7,8]. The DRG may be a key structure mediating part of the pathogenesis of conditions such as fibromyalgia and PLP [1,6,7]. Significant evidence is found in support of the notion that fibromyalgia and PLP involve pathological processes in the periphery [1,6,8,9]. One may recall how peptic ulcer disease was once considered to be a vicious psychosomatic condition [87] until it was found that certain antibiotics are highly effective in many cases. Perhaps clarithromycin affects the psyche, but it also does more… Exploring alternative peripheral mechanisms and elucidating the link between ECM alterations and chronic pain can potentially lead to more effective treatments.

Within the theoretical framework presented here, myofibroblast stress-shielding and matrix remodeling modifies the biomechanical dynamics of the microenvironment around the neuroma, which may either exacerbate stimuli or stress-shield peripheral nerves from local biotensegrity tension to alter the course of the condition over time. A 2005 survey [2] found that 58% of those with phantom pain report having residual limb pain in addition, and about 50% of all unilateral amputees in the study reported pain in the contralateral non-amputated limb. This finding is unsurprising because each patient may have a unique biotensegral imbalance—for example, due to overcompensation of the contralateral side—so their symptoms would depend on fascial tensile forces and sensory nerve involvement. If pre-existing imbalance was present in the body, surgery might exacerbate tension or relieve it, depending on the location of the imbalance and location of the surgery. Stump pain, contrary to phantom pain, occurs under this framework when pain is induced in the limb but not from the part of the neuroma that sends afferent pain signals along the tract that normally represents the amputated part of the body. If axonal sprouting occurs in the spinal level (or cortical area) adjacent to the amputated level, even distant mechanical stimuli from myofascial trigger points and myofibroblasts would be integrated into the perception of phantom limb sensations and pain. Phantom limb pain’s nature is expected to be dynamic because fascia is dynamic by nature. Above a certain threshold, sensations may become painful for an individual. Changing the skeleton changes the biotensegrity’s topography. Tensegrity geodesic domes are made of many nodes, and to change a node is to change the dome. Limb amputation causes asymmetry of the skeleton and the formation of new bony edges with angles that are not necessarily physiological in their architecture and do not always respect tensegrity dynamics of fascia. Active and latent myofascial trigger points adjacent to the neuroma would affect the tensegrity dynamics and provide further inputs to the perception of pain. Interestingly, it is possible that increases in surface electromyogram recordings and, accordingly, increases in muscle tension, can precede certain types of PLP [88].

As was observed in empirical studies (Section 4.2), mechanical interventions may cause unexplained chronic pain and other medical complications, either locally or even at distant anatomical sites, which cannot be solely explained by psychology or neurology. It is not entirely clear how central sensitization and nerve sprouting explain cases where PLP occurs immediately after surgery, or how proprioceptive memory and other theories explain PLP in children born without a limb, or when it appears de novo many years after amputation. What is more, the evidence for either cortical remapping or the idea of a visual proprioceptive mismatch leading to chronic pain is unsatisfactory. First, the theory of cortical reorganization as an underlying cause of PLP is still under question [16,17]. Second, are blind people inherently resistant to PLP? Here, a different perspective is proposed for the theoretical pathogenesis of phantom pain as a neuro-biomechanical disorder that involves the myofascioskeletal system and the cascade of myofibroblast force generation and ECM remodeling of the biotensegrity system. This might help explain PLP cases, or maybe only a subset of PLP cases, e.g., those who may have accompanying myofascial pathologies that promote or exacerbate pain. The sensation of PLP as occurring in a non-existing part of the limb points to a role for the brain, spine, and the nervous system, and compensatory neuroplastic changes, or cortical rearrangements, as the area representing the amputated part evidently still exists in the primary somatosensory cortex and “homunculus” or “self”. Nevertheless, mechanosensitivity of the residual nerve or neuroma and “radiation” of pain to the non-existing part of the limb, in combination with axonal sprouting, may also contribute to the manifestation of PLP. The mechanism of PLP is likely to be more complex and may involve more than just one organ system, so central and peripheral processes are likely to work in concert. The mechanisms listed so far are not necessarily mutually exclusive. While the etiology is multifactorial, the specific clinical manifestation will depend on multiple factors such as the area, layers, and anatomical structures involved, as well as mechanical and biochemical stimuli and the severity of the abnormality [7]. Ideally, the treatment should be individualized accordingly.

### 6.2. Therapeutic Implications

Trigger point injection may be useful as part of the treatment for persistent post-mastectomy pain [89] and for PLP [65]. However, in amputations of traumatic etiology, it is suggested reasonably that acute nerve damage (destroyed nerve plexuses due to traction or pulling forces) play a larger role in the postamputation pain state, while trigger points have a lesser role in such cases [78]. Biofeedback therapies that promote vasodilation or reduce muscle tension in the residual limb have shown promise in alleviating phantom-limb pain, particularly in patients where peripheral factors play a role [1]. The potential of tensegrity-based treatments for PLP are an unexplored area of research. Preliminary evidence suggests that manual treatment based on tensegrity principles might help improve post-mastectomy pain by aiming to relieve the tension in surrounding muscles and the soft tissues that are in direct and indirect contact with the affected area, though clinical trials examining this are lacking [90]. Treatment based on tensegrity principles [91] may be effective in improving chronic shoulder pain when measured at 1 month post treatment [92]. A needling therapy that aims to release fascial tension in local soft tissue improves myofascial trigger point related pain when examined two weeks after treatment [93]. The myofascial biotensegrity-based model posits that a therapy that adheres to tensegrity principles (with the aim of decreasing the pre-stress and imbalance of the overall myofascioskeletal system) might be an effective treatment for fibromyalgia-type symptoms [7] by a tensegrity-based mechanism independent of the gate control theory. This might be relevant for certain cases of persistent post amputation pain, although at this point, it remains a hypothesis. In light of this model, treatments that integrate a tensegrity framework are another therapy worth considering, that differ from existing techniques. Existing therapies such as manipulative therapy, massage, or other manual therapies, typically do not aim at reducing the rigidity of the ECM or target myofibroblasts specifically, and they do not necessarily take into consideration the concept of tensegrity.

Despite attempts at developing therapy for phantom pain, none of the current treatments for PLP are much effective, and by and large fail to employ and leverage the mechanisms of current phantom pain theories [1,13]. Local anesthesia, neurostimulation, cordotomy and rhizotomy, and pharmacological interventions such as anticonvulsants, antidepressants, barbiturates, muscle relaxants, and neuroleptics, have been attempted but show limited benefit [1]. Neurocentric treatments, such as visual-based therapies for PLP were shown to have some effectiveness in some studies [94], a testimony that the central nervous system’s role should not be overlooked even in a peripheral ECM theory. Mirror therapy, a highly popular cognitive treatment according to expert consensus surveys [95], and targeted neuroma interventions have shown partial success for certain patients in part of the studies, indicating the value of these approaches despite the ongoing challenges and suboptimal overall treatment landscape. More recent systematic reviews found no difference in PLP outcomes between mirror therapy and various control therapies [95]. Several studies, including large surveys of patients with amputations, have shown that most treatments for PLP are ineffective and do not take account of the mechanisms underlying the production of the pain [1]. There seems to be a discrepancy between the enthusiasm found in research and the skepticism experienced in clinical practice [95].

### 6.3. Means to Test the Theoretical Model

The theory presented here can be tackled from multiple angles to test it empirically.

- One straightforward non-invasive approach would be to use shear wave elastography, or magnetic resonance elastography for more accuracy, which can help measure the stiffness of myofascial tissue and to compare patients and healthy controls. Compared to control subjects, fibromyalgia/PLP patients should have increased values as measured with shear wave/magnetic resonance elastography, not only at the specific clinical tender spots, but diffusely. Pain is an unpleasant sensory and emotional experience associated with, or resembling that associated with, actual or potential tissue damage (according to the definition of the International Association for the Study of Pain). We would not necessarily expect the area with pain to be strictly correlated only with measured tissue stiffness because pain is the sum of multiple factors, starting from the nerve itself, its microenvironment, the density of innervation, tethering or entrapment, inhibitory pathways, and all the way up to the brain and consciousness. We would, however, expect to find some correlation between imbalance in the overall biotensegrity structure and pain, and to find a correlation between improvement in the overall measured stiffness/imbalance and improvement in clinical complaints, focusing on cases where other known pathologies have been excluded. While the clinical picture may draw attention to a certain anatomical location or tender spot or to a limb, it does not mean the overall pathology is necessarily reflected in this way. Pain, perhaps not intuitive at first, might in certain cases reflect a weak “node” in the tensegrity “dome” being influenced by tension/taut bands/contractures from elsewhere. Each patient can have a different steady state of the biotensegrity system (resulting in a different clinical presentation). We would also expect to find a close causal association between substrate stiffness of ECM and neuroma activity, or between myofibroblast aberrant mechano-activity and neuroma neuroactivity.
- Biopsies can be taken (e.g., subcutaneous tissue, perimysium, perineurium, periosteum) to investigate whether there is an increase in myofibroblast density, or if cells express higher levels of smooth muscle actin or other proto/myofibroblast markers. In muscles, numbers of-SMA–positive cells per 100 myofibers should be higher when compared to control subjects. Due to tensegrity dynamics, a taut band or any area with myofibroblasts might be stress shielded thus causing pain in a more distant region that seems innocent upon focal inspection. But since myofibroblast can de-differentiate and leave behind a remodeled dysfunctional fascia, testing only by this method might actually be deceptive. Beyond cell numbers, immunohistochemical analysis can characterize the deposition and organization of key ECM macromolecules such as collagen I, collagen III, fibronectin, and proteoglycans, assessing changes in fiber alignment and cross-linking that contribute to increased rigidity. cryo-electron tomography can be used to visualize ECM.
- Overall, the secretory profile of myofibroblasts should be altered correspondently, reflecting an overactive state under higher substrate stiffness. Classic systemic markers are not easy to make out because the mechanism is not endocrine or blood-mediated in the main, and there is no leukocyte-driven overt inflammation. In addition, this model has an inherent variability in terms of which anatomical structures are involved. Transcriptomic and proteomic analyses could reveal altered gene and protein expression profiles indicative of an overactive mechanotransduction cascade, involving pathways like YAP/TAZ, RhoA/ROCK, and TGF-β signaling, reflecting higher substrate stiffness.
- Above a certain threshold of substrate stiffness, mechanosignaling would disrupt the intracellular balance between pro-survival/proliferative and apoptotic signals. This is expected to be reflected by gene expression and compensatory mechanisms of myofascial fibroblasts.
- Invasive interventions are expected to affect PLP disease course, especially if operating on the path of the myofascial chains that are most relevant. A treatment that respects principles of tensegrity might be effective in treating PLP, though this depends on whether and in which manner a neuroma is involved. Neuromas complicate the sensory experience. The focus of this paper is less with amputations of traumatic etiology where it is suggested [78] that acute nerve damage (destroyed nerve plexuses due to traction or pulling forces) play a larger role in the postamputation pain state. Nevertheless, neuroma pain and myofascial pain can co-occur.
- Measuring muscle damping should reflect increased muscle tension rather than spasticity. Pendulousness of the limbs of patients compared to age, sex, and body-mass index matched controls could be a simple clinical test to start with.
- Measuring intramuscular pressure via a pressure gauge has been used in studying fibromyalgia [8]. Studying multiple muscles of persons ranked high on the Fibromyalgia Impact Questionnaire score might be insightful; however, the use of invasive measurement tools would alter the measured result and not be ideal for patient safety reasons. Needling multiple sites without respecting tensegrity principles is expected to alter the tensegrity structure and exacerbate the abnormality.
- Traction force microscopy can be used to quantify cellular forces exerted on substrates.
- Biophysical tests—rheometry, strain elastography, stress-relaxation tests, biaxial testing, compression tests, atomic force microscopy, optical coherence elastography, dynamic mechanical analysis, etc., of fascial/myofascial tissue might be insightful, although these would have to take into account the complexity of the model and possible confounding factors, and control for hypermobility syndrome. Age, sex, pH, temperature, hydration, hyaluronic acid composition, adipocytes, cell phenotype and density, are all variables that may affect the properties of fascia in vivo.

This paper presents a conceptual framework for the paradigm. In order to make specific predictions a mathematical model that simulates the human body in three dimensions with its internal tissues and their biophysical properties is needed. Such a model, though simplified, would have to take into account a framework of biotensegrity and continuum biomechanics, which is not typically performed in current models of the human body and musculoskeletal system. A good next step in studying this theory would be to create such a mathematical model and to test it against empirics. (“Prophecy has been given to the fools and babes”).

Even though biotensegrity is the conceptual framework of the model presented in this work, the model does not depend on an actual biotensegrity relationship of the entire human body or (fascio)musculoskeletal system but rather relies on the existence of myofascial kinetic chains and force transmission to adjacent anatomical structures and muscle groups which may suffice for discussing this myofascial biotensegrity-based model. Tensegrity is integrated into the model as a simplification for the purpose of our discussion. The physiological and immunogenic effects of different hyaluronic acid molecular weights and different cell morphology traits add complexity to this model. Also, myofibroblasts can couple to other cells via connexin 43 gap junctions and affect neighboring cells’ electrochemical processes [53]. Obviously, biology does not separate or segregate itself into distinct medical specialties as we do in our profession, and the body and the mind are one.

## 7. Conclusions

This is a conceptual theory-type manuscript that proposes a shift from purely neurocentric models of PLP to a framework where connective tissue, specifically the extracellular matrix and myofibroblast cells, plays a part in the pathogenesis. This offers a new perspective on a condition with poorly understood mechanisms. The paper introduces a model in which surgical interventions disrupt the biomechanical stability of the fascio-musculoskeletal biotensegrity system, acting as a contributing factor in driving chronic pain, working in concert with peripheral and central neurophysiological mechanisms. This offers a new approach to understanding PLP. By linking PLP to an alternative connective-tissue-based paradigm for fibromyalgia, the manuscript suggests a broader, unifying mechanism for idiopathic chronic musculoskeletal pain syndromes. This connection opens new avenues for research into yet unexplored pathophysiological pathways and potential therapeutic targets.

The conceptual framework of biotensegrity under continuum biomechanics may help us understand and explain complex biological phenomena, from the level of the single cell to its microenvironment, the level of the tissue, organ, and up to the level of the whole organism—its behavior, function, kinematics, and kinesthetics, in health and disease. Exploring new avenues of research to find better treatments for PLP should be embraced especially since current theory-based therapies are unsatisfactory. Is it worthwhile to study fascia, or is it boring collagen and nothing much to research?

An extensive structural contiguity of myofascial tissue allows the human body to form an integrated tensegrity-like tensional system without which movement and function would be flawed and inefficient—this ought not be overlooked by research and clinical practice. Future research is needed to help us understand whether biotensegrity-type properties of ECM are being overlooked in psychosomatics and PLP. In any event, studying this enigmatic condition might lead to a better understanding of the body–mind relationship in human beings.

## Data Availability

No new data were created or analyzed in this study. Data sharing is not applicable to this article.

## Abbreviations

The following abbreviations are used in this manuscript:

α-SMA	alpha-smooth muscle actin
CNS	Central nervous system
CRT	Cortical remapping theory
DRG	Dorsal root ganglion
ECM	Extracellular matrix
PLP	Phantom limb pain
TGF-β1	Transforming growth factor beta 1
YAP	Yes-associated protein
